# Determinates of anemia among Human Immune Deficiency Virus positive children on Anti-retro Viral Therapy in selected health facilities, Northwest Ethiopia: A Case-Control Study

**DOI:** 10.1017/jns.2023.79

**Published:** 2023-08-30

**Authors:** Misganaw Guadie Tiruneh, Fassikaw Kebede Bizuneh

**Affiliations:** 1 Department of Health Systems and Policy, Institute of Public Health, University of Gondar, Gondar, Ethiopia; 2 Department of Public Health, College of Health Sciences, Woldia University, Woldia, Ethiopia

**Keywords:** Anaemia, Antiretroviral therapy, HIV-positive children, Metekel zone, ART, antiretroviral therapy, HAART, highly active antiretroviral therapy, Hgb, haemoglobin, HIV/AIDS, human immunodeficiency virus/acquired immunodeficiency syndrome, MUAC, mid-upper arm circumference

## Abstract

Even though antiretroviral therapy (ART) access for human immunodeficiency virus (HIV)-infected children increased dramatically, anaemia has continued as a challenge regardless of a cluster of differentiation (CD4) count and viral load. Hence, the present study aimed to assess the determinants of iron deficiency anaemia among children living with HIV after the initiation of ART. An institution-based unmatched case–control study was conducted among consecutively selected 712 children on HIV care from 1 September to 30 October 2022 in the Metekel zone. A pre-tested and structured data extraction checklist was used to collect the data. Data were analysed using STATA version 16 software. Binary logistic regression was used to find the association between independent variables and anaemia. The level of statistical significance was declared at a value of *P* < 0⋅05. A total of 712 HIV-positive children (178 cases and 534 controls) were included in this study, with a completeness rate of 98⋅8 %. In multivariable analysis, variables that have a statistically significant association with anaemia were as follows: CD4 count <350 (Adjusted Odds Ratio [AOR] 2⋅76; 95 % CI 1⋅76, 4⋅34), World Health Organization (WHO) clinical stage III (AOR 7⋅9; 95 % CI 3⋅5, 17⋅91) and stage IV (AOR 7⋅8; 95 % CI 3⋅37, 18⋅1), cotrimoxazole prophylaxis therapy (AOR 0⋅5; 95 % CI 0⋅31, 0⋅8) and mid-upper arm circumference (MUAC) ≤11⋅5 mm (AOR 2⋅1; 95 % CI 1⋅34, 3⋅28). The present study found that CD4 count, WHO clinical stage, cotrimoxazole prophylaxis therapy and MUAC were significantly associated with anaemia in children on ART. Therefore, continuous screening of anaemia and nutritional treatment is essential in these patients.

## Introduction

Haematological abnormalities are a common occurrence in individuals diagnosed with human immunodeficiency virus (HIV)^(1,2)^. The World Health Organization (WHO) defines anaemia as a haemoglobin level of <11 g/dl for children <5 years old, <11⋅5 g/dl for children 5–11⋅9 years old and <12 g/dl for children 12–14⋅9 years old after altitude adjustment^(3)^. Packed cell volume (PCV) of <33 % is also considered anaemic according to the WHO definition^(3)^. Anaemia is the most frequently encountered haematological abnormality in patients with HIV and has a significant impact on patients’ clinical outcomes such as reduced quality of life, rapid HIV disease progression and increased death^(4,5)^.

The cause of anaemia in HIV-infected children is multifactorial. HIV can, directly and indirectly, affect the survival and function of haematopoietic stem/progenitor cells (HSPCs) in the bone marrow^(6,7)^. Additionally, the drugs used for antiretroviral therapy (ART), inflammatory mediators released during HIV infection and opportunistic infections could also cause anaemia by affecting the proliferation and differentiation of haematopoietic stem cells during haematopoiesis^(8–10)^. Furthermore, anaemia in HIV-infected children may also be caused by nutrient deficiencies^(8,11)^.

Anaemia is a common comorbidity in HIV-infected children and has a significant impact on disease progression and has been noted as a significant predictor of decreased survival time and death^(8)^. However, the risk of anaemia is reduced after the initiation of highly active antiretroviral therapy (HAART)^(9,10)^. Anaemia is one of the global public health problems that affect more than one-third of the world's population. Anaemia is more prevalent in developing countries, particularly Sub-Saharan Africa, which accounts for >89 % of overall anaemia^(8)^. Although anaemia can occur at any time and at all stages of the life cycle, its prevalence is higher especially in younger preschool-aged children due to the increased demand for iron for fast growth^(4,11)^. It adversely affects the cognitive and physical development of children, which in turn results in significant impairment of work capacity and school educational performance^(4,7,11,12)^.

The global prevalence of anaemia in 2019 was 39⋅8 % in children aged 6–59 months, equivalent to 269 million children with anaemia. The prevalence of anaemia in children under five was highest in the African region, 60⋅2 %^(12)^. According to the 2016 report of the Ethiopian Demographic Health Survey (EDHS), 57 % of children aged 6–59 months in Ethiopia and 43 % in the Benishangul Gumuz region suffered from anaemia^(13)^. The prevalence of anaemia among children living with HIV substantially varies across the world regions. In India, the prevalence of anaemia among children living with HIV was 47⋅1 %^(14)^. A prevalence level of 69⋅1 % was reported in the Democratic Republic of Congo^(15)^. In Ethiopia, a systematic review showed that the overall prevalence of anaemia in children living with HIV on HAART was 22⋅3 %^(16)^. However, the review could not include any evidence from the Benishangul Gumuz region, stating that there was no study examining the determinants of anaemia among children living with HIV. Thus, to our knowledge, evidence was limited on determinants of anaemia among children on HAART in this region. Identifying risk factors for anaemia is essential for developing effective interventions and monitoring anaemia control programmes among HIV-infected children. Besides, knowing the determinants of anaemia will help in the early diagnosis and treatment of anaemia based on its cause. Therefore, the finding of the present study will contribute to policymakers, ART clinic managers and service providers as well as to organisations working on the prevention of HIV/AIDS-related deaths for the improvement of the health status of children living with HIV.

## Materials and methods

### Study area

The present study was conducted in the Metekel zone, Benishangul Gumuz region, located 570 km Northwest of Addis Ababa and Northeast of the regional capital city. This zone has seven woredas including Pawie, Mandura, Wombera, Debati, Bullen, Dangur and Guba, based on the 2007 E.C. Ethiopian census, the total population of the zone was estimated to be 276 367, of which 139 119 were men and 137 248 were women. The majority of 238 752 of the population lives in rural areas and is economically dependent on farming. The Metekel zone has seven hospitals, thirteen health centres, seventy-six health posts^(17)^, and there are eight health facilities which provide ART services. The present study was conducted purposefully in the three selected health facilities in the Metekel zone. The selected health facilities were Pawi General Hospital, Gilgel Beles Health Centre and Felege Selam Health Centre. These health facilities were selected based on earlier HAART endorsement for the catchment population since 2012 for an estimated 2968 catchment population^(18)^.

### Study design

A multi-centre facility-based unmatched case–control study was employed from 1 September to 30 October 2022.

### Study population

All HIV-positive children who have enrolled for ART care among the selected health facilities of the Metekel zone.

### Inclusion criteria

All children aged ≤15 years were enrolled for ART care among the selected health facilities were included.

### Exclusion criteria

HIV-positive children with incomplete baseline Hgb and incomplete medical records were excluded.

### Sample size determination

The sample size for this study was calculated using Epi-info version 7 software by considering the following parameters. We used the proportions of controls among the exposed group (P1 = 32⋅6 %) and the proportions of cases for the exposed group (P2 = −45⋅2 %) from the previous research in the North Wollo zone^(4)^, including control-to-case ratios (*R* 3:1), *Z*_2_ *α*/2 = 0⋅05 % within 95 % CI, Power = 0⋅8 and 1⋅78 (AOR 1⋅79; 95 % CI 1⋅13, 3⋅12). Therefore, we added 10 % incompleteness and the final sample size was found to be 720. Therefore, we selected 720 as the final sample size for our study.

### Sampling technique and procedure

*Cases and controls* were selected from the SMART CARE registration systems of all HIV patients based on a unique ART registration. After the proportional sample was allocated for all health institutions, we screened out those who had no Hgb at the baseline in each health institution, and those who had a baseline Hgb of <11 mg/dl were selected as cases and registered all cases in a similar way. Also, for other categories, we selected children who had Hgb ≥ 11 mg/dl as controls.

*Procedure:* Finally, we selected one case and three controls for all health institutions, and we achieved a total of 180 samples of cases and 540 samples of controls for analysis from the selected health facilities based on consecutive sampling techniques. When one case was selected, three controls were consecutively selected from the listed control files. Similarly, the procedure was continued until we reached our final sample size.

### Data collection procedure

A structured and pre-tested data extraction tool, adapted from a previous similar literature^(4,11,19–22)^, was used to extract the required information from the case and control cards. Before the actual data collection, the prepared checklist of variables was pre-tested in thirty-six case notes of HIV-infected children at Jawi Primary Hospital. A 2-d training was given for two diploma nurse data collectors and for two degree public health officers with the objective of achieving the study outcome and maintaining data confidentiality. An assigned supervisor strictly followed the completeness of the collected data and feedback was given daily.

### Data analysis

The collected data were checked, edited and recoded before entered in a software. The collected data were checked for their completeness, entered into Epi-data version 4.2 and then exported to STATA version 16 for further analysis. Descriptive statistics including frequency, percentage, graph and tables were used to present the result. A binary logistic regression analysis was used to identify significant explanatory variables for the outcome variable. The final analysis was computed using a multivariable logistic regression model. Covariates having <0⋅2 at *P*-values in the bi-variable analysis were fitted to multivariable logistic regression analysis. Finally, statistical significance factors associated with Hgb < 11 mg/dl among HIV-positive children were declared as determinants at a *P*-value of <0⋅05. Collinearity was checked by variance inflation factor (VIF) and there was no multi-collinearity among the exposure variables included in the model. Hosmer–Lemeshow goodness-of-fit test was carried out (*P*-value = 0⋅495), indicating the model fits well.

### Ethical approval and permission for chart review

The study was conducted in accordance with the Declaration of Helsinki and ethical approval to conduct the study was obtained from the Ethical Review Board of Woldia University with a reference number of WDU/IRB 015/2014. Names and unique ART numbers of patients were not included in the checklist. Moreover, the data collectors and the supervisor were health professionals who have work experience in the ART clinic to maintain confidentiality of people living with HIV/AIDS. The information retrieved was used only for the study purpose.

## Result

### Socio-demographic characteristics of the respondents

A total of 712 HIV-positive children (178 cases and 534 controls) charts were reviewed in this study, with a completeness rate of 98⋅8 %. The median age of the participants was 116 months, with an interquartile range (IQR 89–145 months). In total, 376 (52⋅8 %) of participants were females. Most of the participants (15⋅6 % of cases and 53⋅65 % of controls) were urban dwellers. Regarding the marital status of the caregivers, 129 (18⋅12 %) of cases and 367 (51⋅5 %) of controls were married. Regarding the occupation of the caregivers, most of them, 82 (11⋅5 %) of cases and 253 (35⋅5 %) of controls, were merchants. Of all the participants, 85 (11⋅94 %) of cases and 292 (41 %) of controls had both their father and mother alive.

The majority of the cases, 101 (14⋅2 %) and 349 (49 %) of controls lived in a family size of 1–3. In total, 283 (39⋅7 %) of the participants were from poorer families (Table 1).

**Table 1.** Socio-demographic characteristics of HIV-seropositive children after starting HAART and their parents in Metekel zone health facilities, Benshangul Gumuz region, Northwest Ethiopia, 2022

Exposure variable	Responses	Anaemic status	
		Anaemic count (%)	Non-anaemic count (%)
Age of the child at enrolment	5–59 months	38 (5⋅34)	74 (10⋅39)
	60–119 months	54 (7⋅58)	193 (27⋅11)
	≥120 months	86 (12⋅08)	267 (37⋅5)
Sex of the child	Male	96 (13⋅5)	240 (33⋅7)
	Female	82 (11⋅5)	294 (41⋅3)
Residence of the caregiver	Urban	111 (15⋅6)	382 (53⋅65)
	Rural	67 (9⋅41)	152 (21⋅35)
Marital status of the caregiver	Single	21 (2⋅95)	89 (12⋅5)
	Married	129 (18⋅12)	367 (51⋅54)
	Divorced	19 (2⋅67)	61 (8⋅57)
	Widowed	9 (1⋅26)	17 (2⋅39)
Occupation of the caregiver	Farmer	25 (3⋅51)	74 (10⋅39)
	Merchant	82 (11⋅52)	253 (35⋅53)
	Employer	32 (4⋅49)	90 (12⋅64)
	Daily labourer	39 (5⋅48)	117 (16⋅43)
Parental status of the child	Both alive	85 (11⋅94)	292 (41⋅01)
	Paternal orphan	38 (5⋅34)	94 (13⋅2)
	Maternal orphan	23 (3⋅23)	83 (11⋅66)
	Both orphan	32 (4⋅49)	65 (9⋅13)
Family size	<4	101 (14⋅19)	349 (49⋅02)
	4–6	77 (10⋅81)	180 (25⋅28)
	>6	0	5 (0⋅7)
Wealth index	Poor	100 (14⋅04)	303 (42⋅56)
	Middle	20 (2⋅81)	65 (9⋅13)
	Rich	58 (8⋅15)	166 (23⋅31)
Functionality status	Working	137 (19⋅24)	411 (57⋅72)
	Ambulatory	27 (3⋅79)	74 (10⋅39)
	Bedridden	14 (1⋅97)	49 (6⋅88)

### Diseases and drug-related factors of iron deficiency anaemia in children on HAART

The present study showed that most of the caregivers (18⋅5 % of cases and 57⋅7 % of controls) were HIV-positive. The majority, 132 (18⋅5 %) of the cases and 429 (58⋅3 %) of controls were not disclosed about their HIV status. Regarding the viral load, 141 (19⋅8 %) of cases and 415 (58⋅3 %) of controls had >1000 copies of HIV per millilitre of blood. In 114 (16 %) of cases and 142 (20 %) of controls, CD4 count was <350. Regarding the WHO clinical stage of the children, 78 (10⋅9 %) of cases and 209 (29⋅4 %) of controls were in clinical stages III and I, respectively. Almost half, 75 (10⋅5 %) of the cases and 178 (25 %) of controls had a history of opportunistic infections. In total, 149 (21 %) of cases had poor adherence. The majority, 111 (15⋅6 %) of the cases and 297 (41⋅7 %) of controls started HAART before 5 years. The majority, 117 (16⋅4 %) of cases and 361 (50⋅7 %) of controls started HAART at Prevention of Mother to Child Transmission (PMTCT) (Table 2).

**Table 2.** Disease and drug-related characteristics of seropositive children on HAART in the selected health facilities of the Metekel zone, Northwest Ethiopia, 2022

Exposure variable	Responses	Anaemic status	
		Anaemic count (%)	Non-anaemic count (%)
HIV status of the caregiver	Positive	132 (18⋅54)	411 (57⋅72)
	Negative	26 (3⋅65)	65 (9⋅13)
	Unknown	20 (2⋅81)	58 (8⋅15)
Disclosure status	Yes	46 (6⋅46)	105 (14⋅75)
	No	132 (18⋅54)	429 (60⋅25)
Viral load	≤1000	37 (5⋅2)	119 (16⋅71)
	>1000	141 (19⋅8)	415 (58⋅29)
CD4 count	<350	114 (16)	142 (19⋅9)
	≥350	64 (8⋅99)	392 (55)
WHO stage	Stage 1	11 (1⋅54)	209 (29⋅35)
	Stage 2	21 (2⋅95)	172 (24⋅16)
	Stage 3	78 (10⋅96)	80 (11⋅24)
	Stage 4	68 (9⋅55)	73 (10⋅25)
Presence of opportunistic infection	Yes	75 (10⋅53)	178 (25)
	No	103 (14⋅47)	356 (50)
Type of opportunistic infection (*n* 253)	Bacterial pneumonia	20 (7⋅9)	56 (22⋅13)
	Diarrhoea	22 (8⋅7)	52 (20⋅55)
	Tuberculosis	23 (9⋅09)	41 (16⋅2)
	Others^a^	10 (3⋅95)	29 (11⋅46)
Duration on ART	≤5 years	67 (9⋅41)	237 (33⋅29)
	>5 years	111 (15⋅59)	297 (41⋅71)
Side effect	Nausea	28 (13⋅27)	46 (21⋅8)
	Headache	17 (8⋅06)	36 (17⋅02)
	Fatigue	9 (4⋅27)	24 (11⋅37)
	Rash	10 (4⋅74)	19 (9)
	Vomiting	4 (1⋅9)	11 (5⋅2)
	Others^b^	3 (1⋅42)	4 (1⋅9)
Treatment failure	Yes	21 (2⋅95)	23 (3⋅23)
	No	157 (22⋅05)	511 (71⋅77)
Adherence	Good	29 (4⋅07)	324 (45⋅5)
	Poor	149 (20⋅9)	210 (29⋅5)
On Isoniazid prophylaxis therapy	Yes	61 (8⋅57)	372 (52⋅25)
	No	117 (16⋅43)	162 (22⋅75)
On cotrimoxazole therapy	Yes	74 (10⋅39)	8 (47⋅47)
	No	104 (14⋅61)	196 (27⋅53)
HAART at PMTCT	Yes	117 (16⋅43)	361 (50⋅7)
	No	61 (8⋅57)	173 (24⋅3)

a Meningitis, Pneumo-Carpus Pneumonia Kaposi sarcoma, acute otitis media, skin dermatitis.

b Unspecified.

### Nutrition-related characteristics of children on HAART

Almost two-thirds, 446 (63 %) of participants had mid-upper arm circumference (MUAC) of >11⋅5 mm, and caregivers of 112 (15⋅7 %) cases got dietary counselling. Ninety-five (13⋅34 %) of cases and 328 (46 %) of controls had normal height-for-age. Thirty-one (4⋅4 %) of cases were underweight (Table 3).

**Table 3.** Nutrition-related characteristics of seropositive children on HAART in the selected health facilities of the Metekel zone, Northwest Ethiopia, 2022

Exposure variable	Responses	Anaemic status	
		Anaemic count (%)	Non-anaemic count (%)
MUAC	≤11⋅5 mm	98 (13⋅76)	168 (23⋅6)
	>11⋅5 mm	80 (11⋅24)	366 (51⋅4)
Dietary counselling for caregiver	Yes	112 (15⋅73)	347 (48⋅74)
	No	66 (9⋅27)	187 (26⋅26)
Admission history due to SAM	Yes	37 (5⋅2)	66 (9⋅27)
	No	141 (19⋅8)	468 (65⋅73)
Stunting Height-for-Age (HFA)	Normal HFA	95 (13⋅34)	328 (46⋅07)
	Moderate stunting	47 (6⋅6)	105 (14⋅75)
	Severe stunting	36 (5 ⋅06)	101 (14⋅19)
Wasting Weight-for-Height (WFH)	Normal WFH	130 (18⋅26)	398 (55⋅9)
	Moderate wasting	27 (3⋅79)	87 (12⋅2)
	Severe wasting	21 (2⋅95)	49 (6⋅88)
Underweight Weight-for- Age (WFA)	Normal WFA	121 (16⋅99)	362 (50⋅84)
	Moderate underweight	26 (3⋅65)	117 (16⋅43)
	Severe underweight	31 (4⋅35)	55 (7⋅72)

### HAART regimen of HIV-positive children in Metekel

**Fig. 1.**
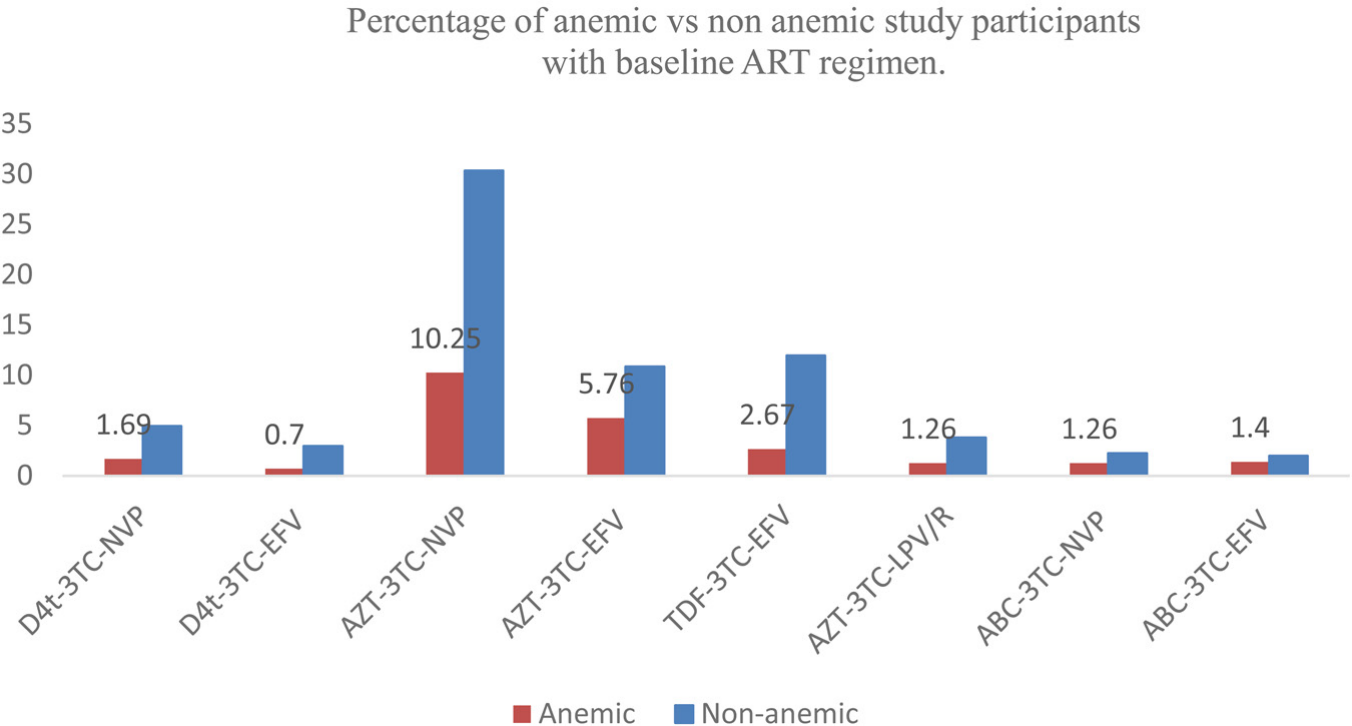
Percentage of anaemic *v.* non-anaemic study participants with a baseline ART regimen of seropositive children on HAART in the selected health facilities of the Metekel zone, Northwest Ethiopia, 2022.

Most of the cases were taking an Zidovudine-based regimen of HAART (Fig. 1).

### Regression analysis on determinants associated with iron deficiency anaemia among HIV-seropositive children on HAART

In bi-variable logistic regression analysis, age of the child at enrolment, sex of the child, residence of caregiver, marital status of the caregiver, parental status of the child, disclosure status, CD4 count, WHO clinical stage, presence of opportunistic infection, duration on HAART, treatment failure, adherence to HAART, on isoniazid prophylaxis therapy, on cotrimoxazole therapy and MUAC of the children had an association with anaemia.

Then, these variables were entered into a multivariable logistic regression to control the confounding effect of one variable on the other predictor variable. In the final model of the multivariable logistic regression analysis, CD4 count, WHO clinical stage, cotrimoxazole preventive therapy and MUAC of the child were the significant factors associated with anaemia among seropositive children after starting HAART.

Accordingly, the odds of being anaemic among HIV-positive children on HAART who had <350 CD4 count were 2⋅76 times higher than those who had CD4 count of ≥350 (AOR 2⋅76; 95 % CI 1⋅76, 4⋅34). Similarly, children who were in WHO clinical stages III and IV were around eight (AOR 7⋅9; 95 % CI 3⋅5, 17⋅91) and (AOR 7⋅8; 95 % CI 3⋅37, 18⋅1) times higher likelihood of being anaemic than those who were in WHO clinical stage I, respectively. Concerning the cotrimoxazole prophylaxis therapy (CPT), children who were taking cotrimoxazole prophylaxis had a 50 % (AOR 0⋅5; 95 % CI 0⋅31, 0⋅8) lower likelihood of being anaemic than their counterparts.

Moreover, MUAC of the seropositive children was found to be significantly associated with anaemia among children on HAART. The odds of being anaemic among HIV-positive children on HAART whose MUAC ≤ 11⋅5 mm was about two times (AOR 2⋅1; 95 % CI 1⋅34, 3⋅28) higher than those who had MUAC > 11⋅5 mm (Table 4).

**Table 4.** Bi-variable and multivariable analysis on predictors of iron deficiency anaemia among children on HAART in the selected health facilities of the Metekel zone, Benshangul Gumuz region, Northwest Ethiopia, 2022

Exposure variable	Responses	Anaemic status		Bi-variable and multivariable analysis		
		Anaemic count (%)	Non-anaemic count (%)	Crude odds ratio (95 % CI)	AOR (95 % CI)	*P*-value
Age of the child at enrolment	5–59 months	38 (5⋅34)	74 (10⋅39)	1⋅59 (1⋅00, 2⋅53)	0⋅73 (0⋅38, 1⋅39)	0⋅344
	60–119 months	54 (7⋅58)	193 (27⋅11)	0⋅86 (0⋅58, 1⋅28)	0⋅84 (0⋅51, 1⋅37)	0⋅489
	≥120 months	86 (12⋅08)	267 (37⋅50)	1	1	
Sex of the child	Male	96 (13⋅5)	240 (33⋅70)	1		
	Female	82 (11⋅5)	294 (41⋅30)	0⋅69 (0⋅49, 0⋅98)	0⋅62 (0⋅4, 1⋅02)	0⋅351
Caregiver residence	Urban	111 (15⋅6)	382 (53⋅65)	1		
	Rural	67 (9⋅41)	152 (21⋅35)	1⋅52 (1⋅06, 2⋅16)	0⋅99 (0⋅60, 1⋅63)	0⋅99
Marital status of the caregiver	Single	21 (2⋅95)	89 (12⋅50)	1		
	Married	129 (18⋅12)	367 (51⋅54)	1⋅48 (0⋅88, 2⋅49)	1⋅39 (0⋅71, 2⋅72)	0⋅334
	Divorced	19 (2⋅67)	61 (8⋅57)	1⋅32 (0⋅65, 2⋅66)	1⋅77 (0⋅72, 4⋅35)	0⋅212
	Widowed	9 (1⋅26)	17 (2⋅39)	2⋅24 (0⋅87, 5⋅72)	0⋅46 (0⋅14, 1⋅48)	0⋅197
Parental status of the child	Both alive	85 (11⋅94)	292 (41⋅01)	1		
	Paternal orphan	38 (5⋅34)	94 (13⋅20)	1⋅38 (0⋅88, 2⋅17)	1⋅17 (0⋅64, 2⋅14)	0⋅588
	Maternal orphan	23 (3⋅23)	83 (11⋅66)	0⋅95 (0⋅56, 1⋅60)	1⋅28 (0⋅65, 2⋅52)	0⋅472
	Both orphan	32 (4⋅49)	65 (9⋅13)	1⋅69 (1⋅03, 2⋅75)	0⋅83 (0⋅39, 1⋅74)	0⋅626
Disclosure status	Yes	46 (6⋅46)	105 (14⋅75)	1		
	No	132 (18⋅54)	429 (60⋅25)	0⋅7 (0⋅47, 1⋅04)	0⋅97 (0⋅57, 1⋅65)	0⋅923
CD4 count	<350	114 (16)	142 (19⋅90)	4⋅91 (3⋅42, 7⋅05)	2⋅76 (1⋅76, 4⋅34)	**0⋅000**
	≥350	64 (8⋅99)	392 (55)	1		
WHO clinical stage	Stage 1	11 (1⋅54)	209 (29⋅35)	1		
	Stage 2	21 (2⋅95)	172 (24⋅16)	2⋅32 (1⋅08, 4⋅94)	1⋅89 (0⋅84, 4⋅23)	0⋅121
	Stage 3	78 (10⋅96)	80 (11⋅24)	18⋅52 (9⋅36, 36⋅63)	7⋅92 (3⋅5, 17⋅91)	**0**⋅**000**
	Stage 4	68 (9⋅55)	73 (10⋅25)	17⋅69 (8⋅87, 35⋅30)	7⋅8 (3⋅37, 18⋅10)	**0**⋅**000**
Presence of opportunistic infection	Yes	75 (10⋅53)	178 (25)	1⋅45 (1⋅02, 2⋅06)	0⋅42 (0⋅23, 1⋅12)	0⋅135
	No	103 (14⋅47)	356 (50)	1		
Duration on ART	≤5 years	67 (9⋅41)	237 (33⋅29)	1		
	>5 years	111 (15⋅59)	297 (41⋅71)	1⋅32 (0⋅93, 1⋅87)	1⋅41 (0⋅89, 2⋅23)	0⋅136
Treatment failure	Yes	21 (2⋅95)	23 (3⋅23)	2⋅97 (1⋅60, 5⋅51)	2⋅58 (1⋅02, 6⋅55)	**0**⋅**045**
	No	157 (22⋅05)	511 (71⋅77)	1		
Adherence	Good	29 (4⋅07)	324 (45⋅50)	1		
	Poor	149 (20⋅90)	210 (29⋅50)	7⋅92 (5⋅13, 12⋅23)	1⋅72 (0⋅93, 3⋅17)	0⋅082
On Isoniazid prophylaxis therapy	Yes	61 (8⋅57)	372 (52⋅25)	0⋅23 (0⋅16, 0⋅33)	0⋅39 (0⋅24, 0⋅64)	**0**⋅**000**
	No	117 (16⋅43)	162 (22⋅75)	1		
On cotrimoxazole therapy	Yes	74 (10⋅39)	8 (47⋅47)	0⋅41 (0⋅29, 0⋅58)	0⋅5 (0⋅31, 0⋅80)	**0**⋅**004**
	No	104 (14⋅61)	196 (27⋅53)	1		
MUAC	≤11⋅5 mm	98 (13⋅76)	168 (23⋅60)	2⋅67 (1⋅88, 3⋅77)	2⋅1 (1⋅34, 3⋅28)	**0**⋅**001**
	>11⋅5 mm	80 (11⋅24)	366 (51⋅40)	1		
Underweight Weight-for- Age (WFA)	Normal WFA	121 (16⋅99)	362 (50⋅84)	1		
	Moderate underweight	26 (3⋅65)	117 (16⋅43)	0⋅66 (0⋅41, 1⋅06)	0⋅4 (0⋅20, 1⋅24)	0⋅081
	Severe underweight	31 (4⋅35)	55 (7⋅72)	1⋅68 (1⋅03, 2⋅74)	0⋅73 (0⋅36, 1⋅45)	0⋅374

*P*-value less than 0.05.

## Discussion

Anaemia is a common comorbidity in HIV-infected children and has a significant impact on disease progression and has been noted as a significant predictor of decreased survival time and death^(4)^. Children are more susceptible to the complications of anaemia due to high requirements, low intake of iron from foods and frequent episodes of infection^(3)^.

The present study found that CD4 count, WHO clinical staging, cotrimoxazole prophylaxis and MUAC were significant predictors of anaemia among seropositive children after starting HAART.

The odds of being anaemic among HIV-positive children on HAART who had <350 CD4 count was 2⋅76 times higher than those who had a CD4 count of ≥350 (AOR 2⋅76; 95 % CI 1⋅76, 4⋅34). In agreement with this finding, a study at Zewditu memorial hospital reported that patients who had a CD4 count of <350 had a high risk of developing anaemia^(21)^. A similar study conducted at the University of Gondar specialised hospital^(20)^ and Burkina Faso^(23)^ reported that a high CD4 count was associated with reduced risk of anaemia among seropositive children on HAART. The possible reason might be due to good immunological recovery during the course of ART medication in children with a high baseline CD4 count^(20)^. In addition, it might also be explained by opportunistic infections and HIV-associated myelosuppression with advanced disease^(24)^, or it might be due to lyses decreased production of RBC resulting in low Hgb with the advancement of HIV-related diseases^(25)^. Research works also revealed that a high baseline CD4 cell count was positively associated with a better immunological response in HIV-infected patients who are on HAART^(26)^. This implies that maintaining a high CD4 count through HAART is crucial to prevent anaemia in HIV-positive children Similarly, children who were in WHO clinical stages III and IV were around eight (AOR 7⋅9; 95 % CI 3⋅5, 17⋅91) and (AOR 7⋅8; 95 % CI 3⋅37, 18⋅1) times higher likelihood of being anaemic than those who were in WHO clinical stage I, respectively. The finding of the present study was in line with a study done in Afar^(19)^ and Harar^(22)^. The possible explanation for this might be due to immunologic deterioration when the diseases progress to the advanced stage which causes opportunistic infections. In addition, the advanced stage of HIV disease may also be associated with deficiencies of micronutrients in children, such as vitamin A, which is estimated to have a role in iron transport and erythropoiesis^(27)^. This implies that halting the progress of the diseases through HAART, nutrition and other comprehensive care is one of the important strategies to prevent anaemia among children in HAART.

Besides, children who were taking cotrimoxazole prophylaxis had 50 % (AOR 0⋅5; 95 % CI 0⋅31, 0⋅8) lower likelihood of being anaemic than their counterparts. This study finding was consistent with a study done in Wolaita Sodo^(28)^, which reported that children who were taking cotrimoxazole prophylaxis had a lesser odds of being anaemic compared with those who were not.

Another study done at the University of Gondar specialised hospital also found that CPT prevents anaemia among children on HAART^(11)^. The possible explanation for this might be CPT prevents opportunistic infection, which causes anaemia in children on HAART. Additionally, it might also be due to the cytokine reduction effect of cotrimoxazole that impair erythropoiesis, by reducing immune activation as well as by preventing infection^(29)^, which implies that CPT prevents anaemia in HIV children on HAART.

Furthermore, the odds of being anaemic among HIV-positive children on HAART whose MUAC ≤ 11⋅5 mm was about two times (AOR 2⋅1; 95 % CI 1⋅34, 3⋅28) higher than those who had MUAC > 11⋅5 mm. This study finding was in agreement with the study done in Afar^(19)^ and North Wollo^(4)^, which reported that children who were stunted were more likely to be anaemic than children who were not stunted. A similar study conducted in India reported that stunting and vitamin A deficiency were associated with anaemia^(14)^. This might be due to malnourished children having an association with iron deficiency which is the commonest cause of anaemia. The other reason can be nutritional deficiencies that can contribute to a decrease in the immune response, which in turn causes opportunistic infection and anaemia in HIV-seropositive children. This implies that preventing HIV-positive children from malnutrition is important to prevent children from anaemia.

## Limitations of the study

The data were collected from the records which were not including other causes of anaemia like malaria, intestinal parasites and so on. This study classifies anaemia as Hgb < 11 g/dl which does not consider the age-specific value level of haemoglobin.

## Conclusion

In the present study, CD4 count, WHO clinical stage, CPT and MUAC were significantly associated with anaemia among HIV-positive children on HAART. Early diagnosis and treatment of anaemia by HAART programmes is recommended. We also recommend that the healthcare providers focus on nutritional counselling and management along with HIV care.
